# Endoscopic Resection Versus Laparoscopic Resection for Gastric Submucosal Tumors: A Systematic Review and Meta‐Analysis of Safety and Efficacy

**DOI:** 10.1111/ases.70104

**Published:** 2025-06-24

**Authors:** Kengo Hayashi, Saki Hayashi, Roberto Passera, Chiara Meroni, Rebecca Dallorto, Chiara Marafante, Carlo Alberto Ammirati, Alberto Arezzo, Noriyuki Inaki

**Affiliations:** ^1^ Department of Gastrointestinal Surgery Kanazawa University Kanazawa Japan; ^2^ Department of Surgical Sciences University of Turin Turin Italy; ^3^ Department of Medical Sciences University of Turin Turin Italy

**Keywords:** complete resection rate, EFTR, endoscopic resection, gastric submucosal tumor, laparoscopic resection, meta‐analysis

## Abstract

**Introduction:**

Gastric submucosal tumors (G‐SMTs) vary in malignancy risk, with surgical resection as standard treatment. Although extended endoscopic resection (eER) offers a less invasive option, its outcomes relative to laparoscopic resection (LR) remain unclear. This study evaluates the safety and efficacy of eER and LR.

**Materials and Methods:**

A systematic review and meta‐analysis included articles comparing eER and LR for G‐SMTs. The primary outcome was a complete resection rate.

**Results:**

17 studies involving 1262 eER and 990 LR patients were included. LR showed a higher complete resection rate (RR 0.98, 95% CI 0.97–0.99, *p* < 0.01). eER favored operative time (95% CI ‐57.66 to −23.71, *p* < 0.01), blood loss (95% CI ‐63.46 to −17.45, *p* < 0.01), time to oral intake (95% CI ‐1.64 to −0.33, *p* < 0.01), and hospital stay (95% CI ‐1.75 to −0.13, *p* = 0.023). Subgroup analysis comparing endoscopic full‐thickness resection (EFTR) to LR showed no significant difference in complete resection (RR 0.98, 95% CI 0.95–1.01, *p* = 0.18).

**Conclusions:**

LR may offer a higher complete resection rate, but eER demonstrated better short‐term outcomes. EFTR achieved comparable resection rates to LR, supporting broader adoption with further technical refinement.

## 1 Introduction

Gastric submucosal tumors (G‐SMTs) are relatively common in clinical practice and encompass a wide range of histopathological types, ranging from benign tumors, such as leiomyomas and schwannomas, to malignant neoplasms, most notably gastric gastrointestinal stromal tumors (G‐GISTs) [1]. As it is known, the European Society of Medical Oncology (ESMO) and the National Comprehensive Cancer Network (NCCN) guidelines recommend the complete resection of the tumor without lymphadenectomy as the standard treatment for G‐GISTs [2, 3]. When the treatment is required, surgical resection is the mainstay of it, emphasizing en bloc resection while avoiding capsule rupture to minimize the risk of peritoneal dissemination [4].

Endoscopic resection (ER) was first introduced in 1988 as a treatment for superficial early lesions [5]. However, advancements in endoscopic techniques, improvements in medical devices such as endoscopic ultrasound (EUS), and the development of novel methods such as titanium clipping and the nylon rope technique have significantly expanded its applications [6]. These innovations have facilitated the progression of ER, making endoscopic full‐thickness resection (EFTR) or other extended ER (eER) a feasible approach for gastric lesions.

Despite reports demonstrating its feasibility, concerns remain regarding the safety and clinical outcomes of eER, with serious complications and the need for emergency surgical conversion still being reported [4]. Moreover, there is limited evidence directly comparing the therapeutic outcomes of eER and surgical resection for the treatment of G‐SMTs, particularly in terms of complete resection rates. Consequently, eER is not yet considered a standard treatment for G‐GISTs, and its role remains controversial. However, as a less invasive alternative to laparoscopic resection (LR), eER holds promise, and further research is needed to establish robust evidence supporting its efficacy for G‐SMTs.

Some meta‐analyses have already been conducted to compare the outcomes of eER and LR for gastric G‐GISTs, with the most recent published in 2022 [7, 8]. That meta‐analysis concluded that ER yielded perioperative and oncological outcomes comparable to or better than those of LR. Additionally, the ER group in that study included all types of endoscopic procedures as a single category, without stratification by specific techniques. Since then, five additional studies—including two that employed propensity score matching—have been published, some of which reported findings inconsistent with the earlier meta‐analysis. Given these recent developments and the methodological limitations of previous analyses, we conducted an updated meta‐analysis that incorporates these newer studies and separately evaluates outcomes by endoscopic technique, thereby providing a more comprehensive and clinically relevant comparison of eER and LR for G‐SMTs.

Furthermore, even in G‐SMTs without a definitive diagnosis of G‐GISTs, resection is often considered for cases presenting with symptoms, progressive enlargement over time, tumor size exceeding 5 cm, or features suggestive of malignancy. To address these considerations and accumulate a broader dataset, we expanded our meta‐analysis to include all types of G‐SMTs, provided that the necessary outcomes for this study could be extracted. To date, no meta‐analysis has systematically compared eER and LR specifically for G‐SMTs. Therefore, this study represents the first comprehensive meta‐analysis to evaluate and compare the short‐term outcomes of eER and LR across all types of G‐SMTs. Through this approach, our study aims to provide a clearer understanding of the potential role of eER in the treatment of G‐SMTs and to assess whether it can be considered a viable alternative to surgical resection for these tumors.

## 2 Materials and Methods

The approach to the analysis and development of inclusion criteria was meticulously performed, adhering closely to the guidelines outlined in the Cochrane Handbook for Systematic Reviews of Interventions, as well as the standards established by the Preferred Reporting Items for Systematic Reviews and Meta‐Analyses checklist (PRISMA 2020) [9, 10] (File S1).

### 2.1 Eligibility Criteria

According to population, interventions, comparators, outcome measures, and setting (PICOS) criteria, we selected studies for our review that met the following inclusion criteria: (1) articles comparing endoscopic versus laparoscopic resection for gastric submucosal tumors without including laparoscopy endoscopy cooperative surgery; (2) that reported any of the variables of interest; (3) in the case of duplicate studies, the largest and complete reports were included; and (4) studies with more than ten patients per study arm were included. Additionally, (5) unless accompanied by detailed tables enabling extraction of essential background information and relevant outcomes, conference abstracts were excluded owing to insufficient data. In this study, as the primary endpoint was the complete resection rate of tumors, submucosal tumors of the stomach were included regardless of histological type, whereas tumors involving the duodenum or esophagus were excluded. To maximize data acquisition and minimize bias, gray literature, including conference abstracts, was incorporated when relevant information could be extracted. In the propensity score‐matched study, only the data of patients following propensity score adjustment was utilized. Additionally, all secondary analyses, such as meta‐analyses or systematic reviews, were examined to identify any potentially overlooked articles not included in the primary analysis.

### 2.2 Outcomes

The primary outcome of this study was a complete resection rate. A complete resection was defined as a resection in which no residual tumor was identified in the pathological examination. Secondary outcomes were procedural time, blood loss, time to oral intake, length of hospital stays, completion rate, postoperative complications, and postoperative infectious complications. The completion rate indicated the proportion of procedures that were performed as planned. Incompletion of the procedure was considered to be conversion to surgery in the eER group and to open surgery in the LR group. Additionally, subgroup analysis was conducted for the EFTR group extracted from the eER group for the primary outcome.

### 2.3 Literature Search Strategy

The search strategy was developed in OVID Medline, and then its final optimized version was translated to Embase and Cochrane CENTRAL, and relevant articles were searched in July 2024. The keywords used included: “Gastric,” “Gastrointestinal Stromal Tumor,” “Neurilemmoma,” “Schwannoma,” “Leiomyoma,” “Lipoma,” “Neurofibroma,” “Neuroendocrine Tumor,” “Lymphoma,” “Ectopic pancreas,” “Submucosal Tumor,” “Subepithelial Tumor,” “Endoscopic(al) resection,” “Laparoscopic resection,” “Endoscopic(al) Dissection,” “Endoscopic(al) excavation,” “Endoscopic(al) mucosectomy.” All relevant full‐text or conference abstracts of randomized controlled trials (RCTs) and observational studies (retrospective/prospective) were included. We selected only articles written in English.

### 2.4 Literature Screening and Data Extraction

Titles were initially screened, and abstracts of potentially relevant studies were reviewed. After full‐text evaluations, the following data were collected: authorship, publication year, country, type of publication (articles or abstracts), study design, recruitment period, patient count, gender, age, body mass index (BMI), procedure performed, tumor pathology/location/size, complete resection rate, and completion rate. All screening and data extraction procedures were independently conducted by five authors (KH, SH, CMe, CMa, RD, CAA). To ensure consistent application of inclusion/exclusion criteria among all reviewers and minimize bias during the screening process, an inter‐rater agreement test was performed. This test involved all reviewers screening the same 10% of randomly selected articles and discussing any disagreements before proceeding with the full screening. This test helped minimize criteria differences between reviewers, ensuring alignment on inclusion/exclusion decisions. A second round of the inter‐rater agreement test was planned in case of a high number of disagreements; however, it was not necessary this time as there was little discrepancy among the raters.

### 2.5 Quality Assessment

The methodological quality and risk of bias of each study were determined by all the authors according to the Cochrane Handbook for Systematic Reviews of Interventions v. 6.4 (August 2023) and the Newcastle‐Ottawa Scale (NOS) [11]. The NOS scores were assessed by two authors (KH and SH), and all included studies scored 7 or higher, indicating a low risk of bias (File S2).

### 2.6 Statistical Analysis

All analyses were performed according to the original treatment allocation (intention‐to‐treat analysis). For binary outcome data, the relative risks (RR) and 95% confidence intervals (CIs) were estimated using the Mantel–Haenszel method. For continuous outcome data, the mean differences (MD) and 95% CIs were estimated using the inverse variance weighting; when means and/or standard deviations (SDs) were not reported, they were estimated from reported medians, ranges, and sample size as described by Hozo et al. [12]. Heterogeneity was explored by the Baujat plot (showing the contribution of each study to the overall heterogeneity) and assessed by the *I* ^2^ measure of inconsistency, statistically significant if *I* ^2^ > 50%. A fixed‐effects model was at first used in all meta‐analyses, whereas the random‐effects model was used for the outcomes showing *I* ^2^ > 50%. Potential sources of heterogeneity were explored by different sensitivity analyses: comparing fixed‐ vs. random‐effects models (thus incorporating heterogeneity by using the second method); performing a subgroup analysis comparing the complete resection rate between EFTR and LR, checking the results of cumulative (sequentially including studies by date of publication) and influential meta‐analyses (calculating pooled estimates omitting one study at a time). Publication bias was assessed by generating a funnel plot and performing the linear regression test on its asymmetry. Furthermore, when publication bias was detected, the heterogeneity and overall effect were assessed using the Baujat plot. All analyses were conducted in February 2025 by *R* 4.4.1 package meta (www.r‐project.org).

## 3 Results

**FIGURE 1:**
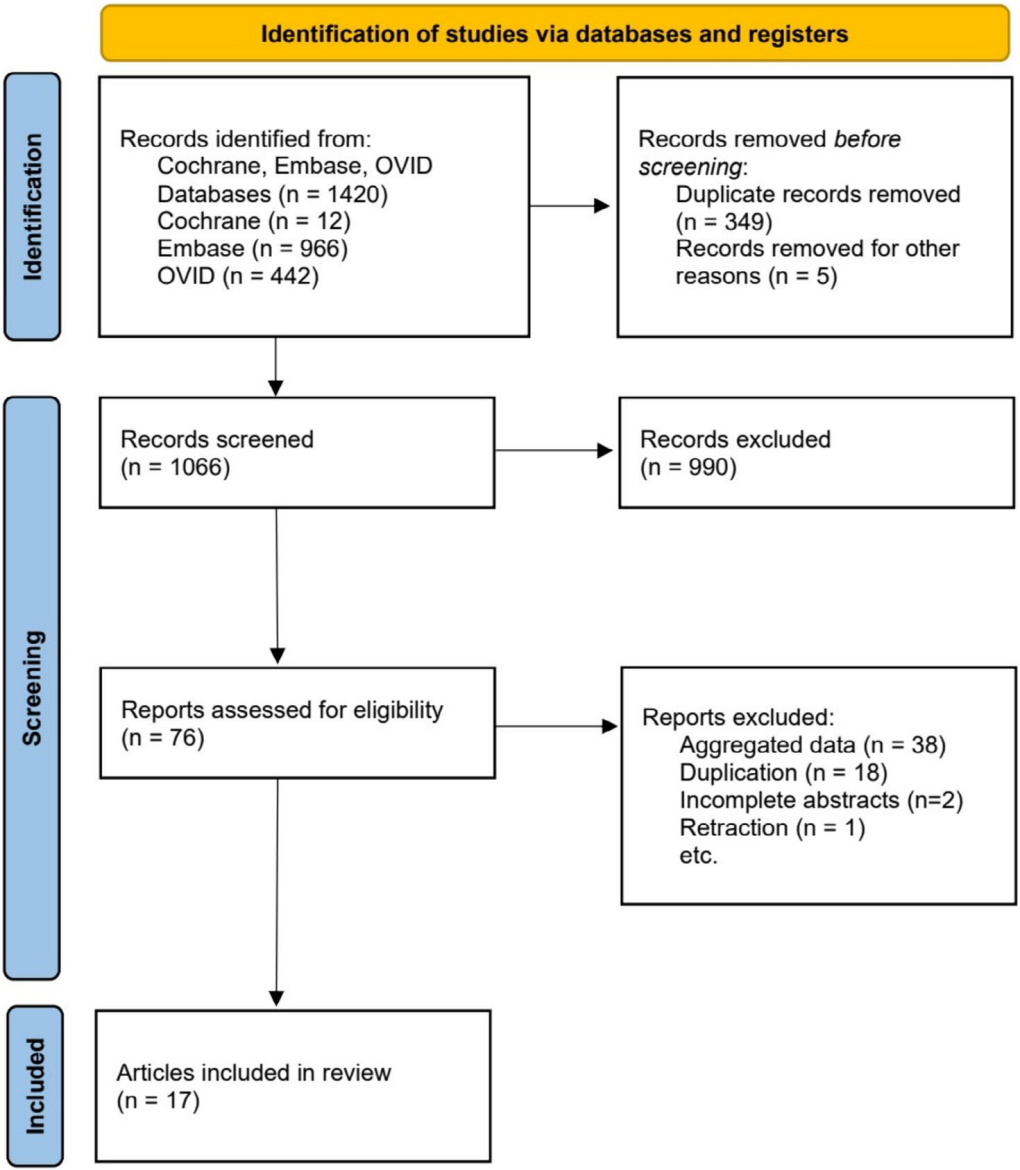
PRISMA flowchart diagram illustrating the systematic search and study selection strategy.

Figure 1 represents The PRISMA flow diagram for this systematic review presented in the Guideline flow diagram. In total, 1420 studies were collected by the initial search. After the removal of duplications and other unsuitable titles, 1066 articles were reviewed by titles and abstracts, and the remaining 78 articles were screened by full‐text reading. Finally, 17 studies met our inclusion criteria and were incorporated into the meta‐analysis, including a total of 2252 patients: 1262 underwent eER (56.0%) and 990 LR (44.0%) [13, 14, 15, 16, 17, 18, 19, 20, 21, 22, 23, 24, 25, 26, 27, 28, 29]. All 17 studies are retrospective observational cohort studies, and 4 of them were analyzed using propensity score matching. The characteristics of the included articles in this study are summarized in Table 1.

**TABLE 1:** Summary of the articles.

Author	Year	Country	Design	Time of recruitment	Treatment	Number of pts	Sex (M)	Age (years)	BMI (kg/m^2^)	Procedure	Pathology	Tumor size (mm)	Tumor site	R0 rate (%)
Wang. L	2011	China	R	2008–2009	ER	66	31	44	na	ESD: 35, EFTR: 31	na	*15* (8–17)	fundus: 38, body: 26, antrum: 2	na
					LR	43	23	41	na	WR: 43	na	*11* (7–17)	fundus: 23, body: 16, antrum: 4	na
Jeong. I	2012	South Korea	R	2002–2007	ER	27	36	55.4 ± 12.4	na	ESD: 27	GIST	*15* (7–45)	na	85
					LR	57				WR: 53, DG: 3, TG: 1, plus LC: 2	GIST	*37* (12–75)	na	98.2
Wu. C	2015	China	R	2009–2014	ER	50	28	44.3	na	EFTR	GIST	34	fundus:14, body: 23, antrum: 13	100
					LR	42	23	47.6	na	WR, IGS, SG	GIST	38	fundus:8, body: 19, antrum: 15	100
Meng. F	2016	China	R	2010–2015	ER	68	28	58.5 ± 7.3	25.1 ± 3.7	ESD: 68	GIST: 49, Leiomyma: 16, Schwannoma: 3	25.8 ± 9.7	cardia: 5, fundus: 27, body: 29, antrum: 7	na
					LR	47	20	57.1 ± 6.9	25.9 ± 4.2	WR: 47	GIST: 31, Leimyoma: 13, Schwannoma: 3	37.1 ± 12.3	cardia: 2, fundus: 20, body: 22, antrum: 3	na
Wang. H	2016	China	R	2011–2013	ER	35	25	55	na	EFTR: 35	GIST: 35	13 ± 5	fundus: 10, body: 15, antrum: 10	100
					LR	33	20	56	na	WR: 33	GIST: 33	16 ± 4	fundus: 12, body: 10, antrum: 11	100
Dai. W	2017	China	R	2011–2014	ER	262	106	*58* (23–81)	na	ESD: 262	GIST	13.3 ± 7.8	fundus: 154, body: 59, antrum: 7, cardia: 34	na
					LR	73	30	*60* (28–83)	na	na	GIST	19.7 ± 9.3	fundus: 61, body: 35, antrum: 21, cardia: 5	na
Gluzman. M	2017	Russia	R	2010–2016	ER	22	na	na	na	ESD: 6, STER: 8, EFTR: 8	GIST	23 ± 3	na	100
					LR	42	na	na	na	WR: 33, SubTG: 4, SR: 3, IGS: 2	GIST	47 ± 8	na	100
He. B	2018	China	R	2012–2016	ER	62	36	51.5 ± 9.3	na	ESD: 62	GIST	34 ± 11	fundus: 19, body: 32, antrum: 11	100
					LR	84	56	53.3 ± 8.72	na	WR: 84	GIST	37 ± 13	fundus: 27, body: 38, antrum: 19	100
Zhang. H	2019	China	R	2013–2017	ER	152	58	55.9 ± 11.8	na	ESD: 6, ESE: 26, EFR: 61	GIST: 91, Leiomyoma: 17, Lipoma: 6, Schwannoma: 5, Neurofibroma: 4, Ectopic pancreas: 20, Neuroendocrine tumor: 7, Lymphoma: 2	19 ± 08	upper: 59, middle: 61, lower: 32	98.7
					LR	123	54	58.5 ± 10.8	na	WR: 93, DG: 30	GIST: 81, Leiomyoma: 15, Lipoma: 2, Schwannoma: 11, Neurofibroma: 4, Ectopic pancreas: 9, Neuroendocrine tumor: 1, Lymphoma: 0	36 ± 12	upper: 23, middle: 87, lower: 13	97.5
Yip. HC	2019	China	R	2012–2018	ER	29	10	53.7 ± 11.5	na	na	GIST: 11, Leiomyoma: 11, Ectopic pancreas: 4, Granular cell tumor: 1, Calcifying fibrous tumor: 1, Schwannoma: 0, Lipoma: 1	31	cardia: 13, greater curve: 3, lesser curve: 3, proximal body: 3, antrum: 7	93.1
					LR	28	8	61.3 ± 11.2	na	na	GIST: 22, Leiomyoma: 0, Ectopic pancreas: 2, Granular cell tumor: 0, Calcifying fibrous tumor: 1, Schwannoma: 3, Lipoma: 0	23	cardia: 1, fundus: 15, lesser curve: 6, proximal body: 5, antrum: 1	100
Dong. X	2020	China	P	2006–2017	ER	45	24	56.3 ± 9.8	22.2 ± 3.4	ESD	GIST: 45	26 ± 7	fundus: 25, body: 13, antrum: 7	97.8
					LR	45	26	55.8 ± 9.9	22.4 ± 4.5	WR, PR	GIST: 45	29 ± 8	fundus: 20, body: 21, antrum: 4	100
Zhao. Y	2020	China	R	2009–2017	ER	85	31	57.0 ± 9.66	na	EFTR: 85	GIST very low risk: 56, low risk: 23, intermediate risk: 3, high risk: 3	16 ± 8.8	cardia: 6, fundus: 55, body: 20, antrum: 4	95.3
					LR	64	29	57.7 ± 10.3	na	WR: 64	GIST very low risk: 11, low risk: 41, intermediate risk: 5, high risk: 7	31.3 ± 11.1	cardia: 3, fundus: 36, body: 18, antrum: 6	100
Li.D	2022	China	P	2011–2018	ER	49	19	58.8 ± 14.1	na	ESD, EFTR	GIST: 49	29.7	cardia: 1, fundus: 34, body: 14	na
					LR	49	31	57.3 ± 16.1	na	WR, Gastrectomy	GIST: 49	29.5	cardia: 1, fundus: 31, body: 16, antrum: 1	na
Li.Y	2022	China	P	2008–2019	ER	27	12	62.2 ± 11.5	na	STER, ESE, EFTR	GIST: 27	33.3 ± 9.45	cardia: 4, fundus: 8, body: 14, antrum: 1	92.6
					LR	27	10	61.1 ± 12.4	na	na	GIST: 27	33.0 ± 8.95	cardia: 1, fundus: 12, body: 13, antrum: 1	96.3
Liu. Y	2022	China	P	2016–2020	ER	94	43	59.6 ± 10.3	na	ESD, EFTR, STER	GIST: 94	29.6 ± 8.2	na	42.5
					LR	94	46	59.6 ± 10.5	na	WR, DG, PG	GIST: 94	28.7 ± 6.2	na	100
Meng. X	2022	China	R	2010–2015	ER	89	40	56.8 ± 9.8	na	ESD: 55, EFTR: 23, STER: 11	GIST: 89	26 ± 5	cardia: 16, fundus: 55, body: 15, antrum: 3	97.8
					LR	45	23	55.0 ± 12.6	na	WR: 33, SG: 12	GIST: 45	29 ± 5	cardia: 8, fundus: 21, body: 14, antrum: 2	100
Chang. W	2023	Taiwan	R	2013–2021	ER	100	*46*	*56* (*49–62*)	na	ESD: 92, STER: 8	GIST: 43, neuroendocrine tumor: 1, benign: 55	na	upper: 81, middle: 6, lower:13	na
					LR	94	*47*	*62* (*52–70*)	na	WR: 92, DG: 2	GIST: 78, neuroendocrine tumor: 1, lipoleiomyosarcoma: 1, benign: 14	na	upper: 61, middle: 14, lower: 19	na

*Note:* ER vs. LR are compared. Data are reported as numbers, mean ± standard deviation, and *median* (*italic*) (range or *interquartile* (*italic*)).

Abbreviations: DG: distal gastrectomy, EFTR: Endoscopic endoscopic Fullfull‐Thickness thickness Resectionresection, ER: Endoscopic resection, ESD: Endoscopic endoscopic Submucosal submucosal Dissectiondissection, ESE: Endoscopic endoscopic Submucosal submucosal Excavationexcavation, IGS: Intragastric surgery, LR: Laparoscopic laparoscopic resection, na: not available, P: propensity score matching, PG: proximal gastrectomy, R: retrospective, STER: Submucosal submucosal TtunnelingTunnelling Endoscopic endoscopic Resectionresection, TG: total gastrectomy, WR: Wedge wedge Resectionresection.

### 3.1 Primary Outcomes

**FIGURE 2:**
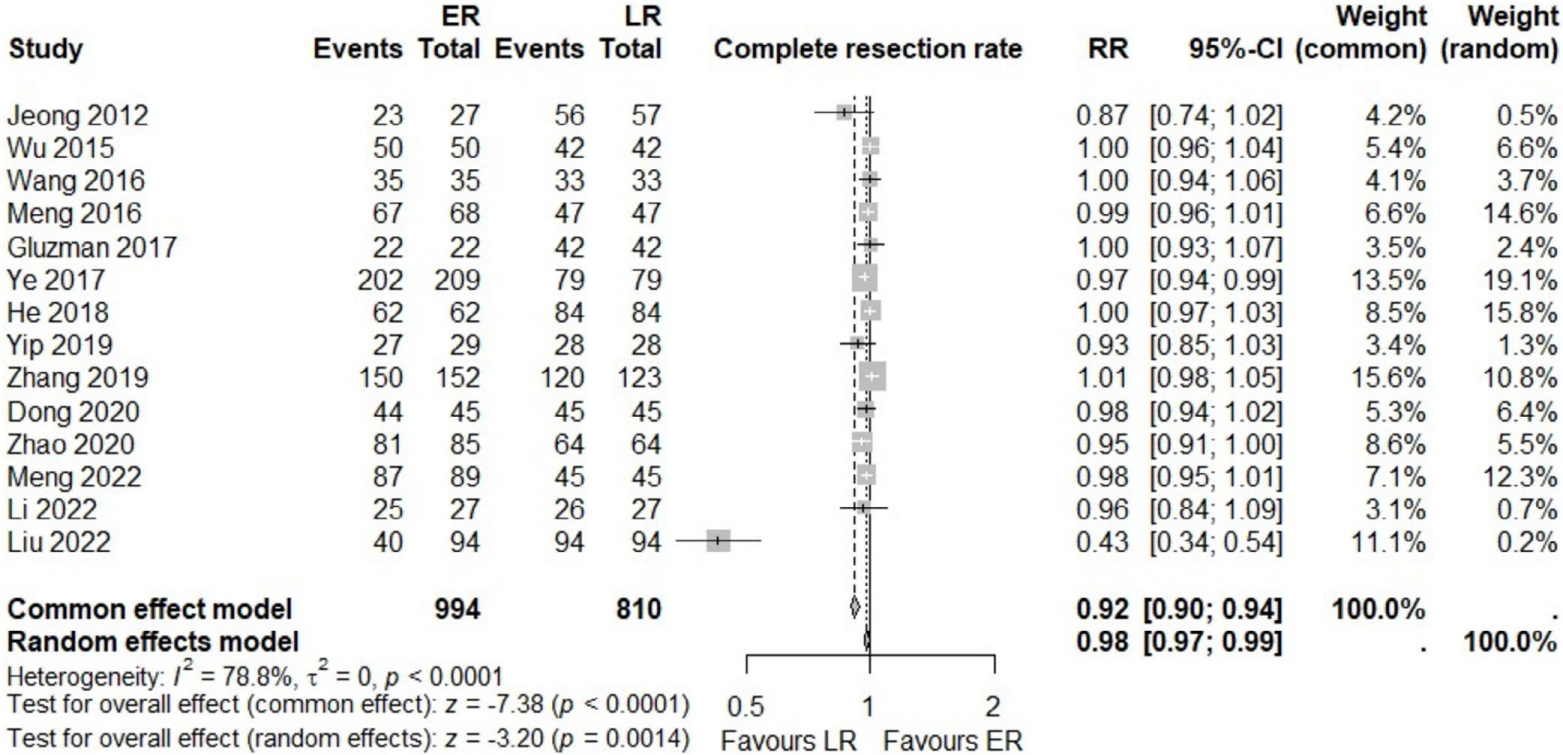
Forest plot for complete resection rate between eER and LR.

We assessed the complete resection rate as the primary outcome of the current meta‐analysis. The complete resection rate was reported in 14 studies, including 994 cases in eER and 810 cases in LR. Owing to the heterogeneity being over 50% (*I* ^2^ = 78.8%), the random‐effects model was chosen for evaluation. The result showed favor in LR with RR of 0.98 (95% CI 0.97; 0.99, *p* = 0.0014) (Figure 2). Egger's test indicated potential publication bias (*p* = 0,0381). In addition, the study contributing to heterogeneity also demonstrated a substantial impact on both heterogeneity and the overall effect in the Baujat plot (File S3).

### 3.2 Secondary Outcomes

**FIGURE 3:**
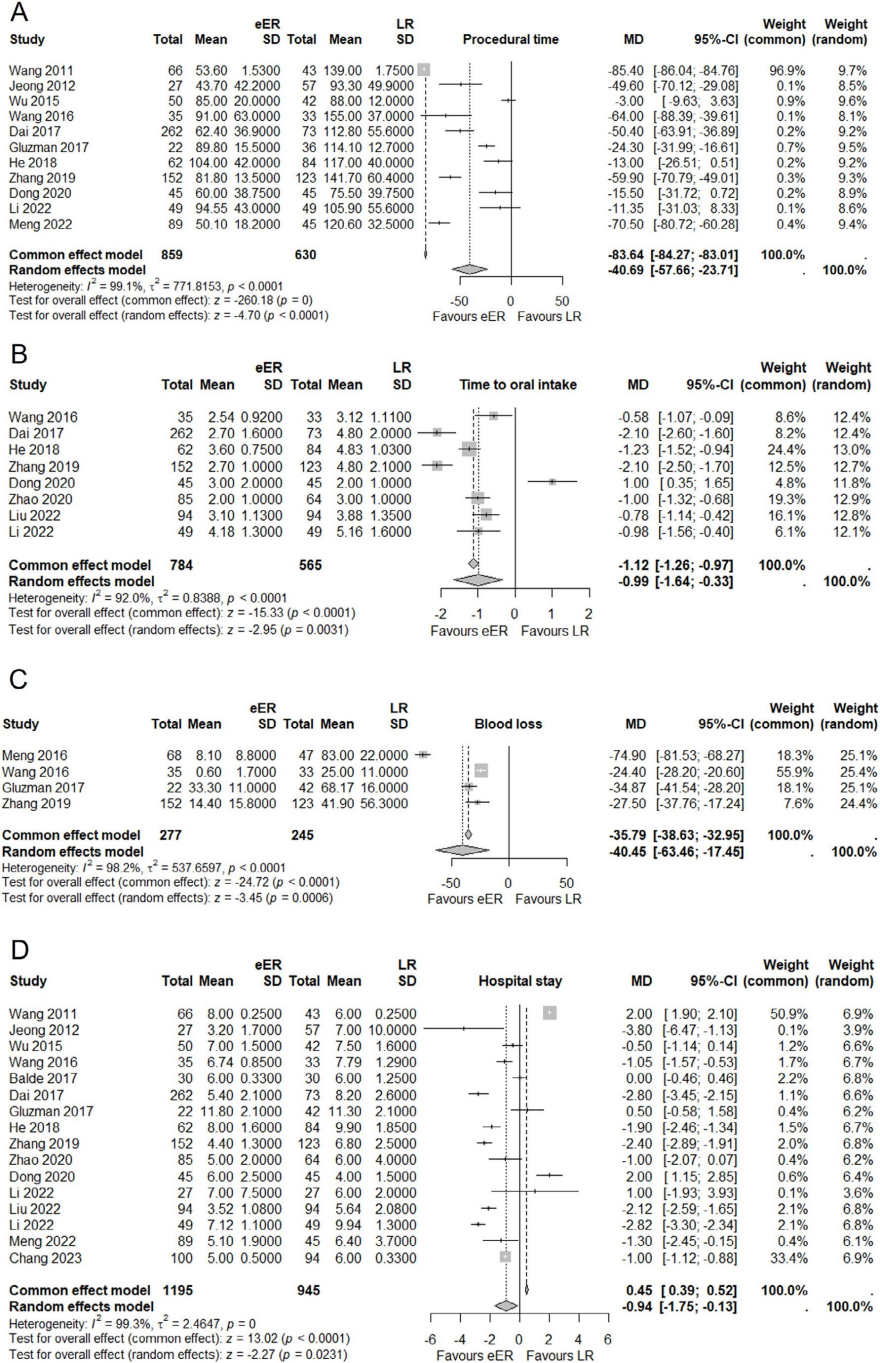
(A) Forest plot for procedural time between eER and LR. (B) Forest plot for blood loss between eER and LR. (C) Forest plot for time to oral intake between eER and LR. (D) Forest plot for hospital stay between eER and LR.

Procedural time was reported in 11 studies, including 859 cases in eER and 630 in LR. We chose the random‐effects model owing to the high heterogeneity (*I* ^2^ = 99.1%), showing favors in eER with MD of −40.69 (95% CI ‐57.66; −23.71, *p* < 0.0001) (Figure 3A). Although heterogeneity was present among the studies, the Baujat plot did not suggest that any single study had a disproportionately large impact owing to publication bias (File S4A).

Blood loss was reported in four studies, including 277 cases in eER and 245 in LR. The random‐effects model was used owing to the high heterogeneity (*I* ^2^ = 98.8%), which showed favor in eER with an MD of −40.45 (95% CI ‐63.46; −17.45, *p* = 0.0018) (Figure 3B).

Time to oral intake was reported in eight studies, including 784 cases in eER and 565 in LR. The random‐effects model was used owing to the high heterogeneity (*I* ^2^ = 92%), which showed favor in eER with MD of −0.99 (95% CI ‐1.64; −0.33, *p* = 0.0031) (Figure 3C). Regarding procedural time, blood loss, and time to oral intake, the number of studies included was insufficient to perform a linear regression test of the funnel plot. However, no study demonstrating both high heterogeneity and a strong overall effect was identified in the Baujat plot (File S4B,C).

The length of hospital stays was reported in 16 studies, including 1195 cases in eER and 945 in LR. Owing to the high heterogeneity (*I* ^2^ = 99.3%), the random‐effects model was chosen for evaluation. The result showed favor in eER with an MD of −0.94 (95% CI ‐1.75; −0.13, *p* = 0.023) (Figure 3D). The Baujat plot indicated high heterogeneity and overall effect in the study by Wang et al. However, excluding this outlier further strengthened the significance of the results, suggesting that its presence did not compromise the overall findings (File S4D).

**FIGURE 4:**
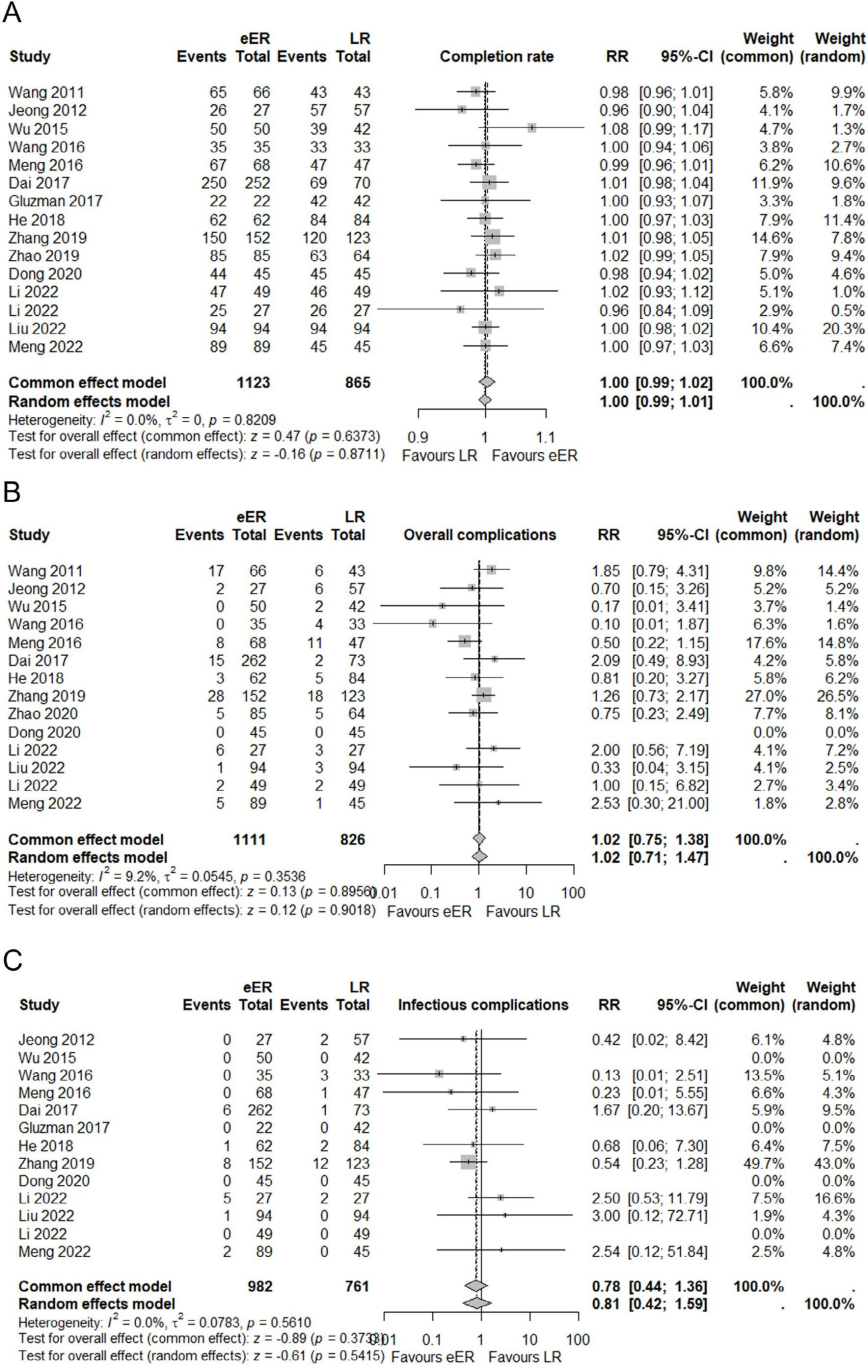
(A) Forest plot for completion rate between eER and LR. (B) Forest plot for overall complication rate between eER and LR. (C) Forest plot for infectious complication rate between eER and LR.

Completion rate was reported in 15 studies, including 1123 cases in eER and 865 in LR. Owing to the extremely low heterogeneity (*I* ^2^ = 0%), the common effect model was chosen for evaluation. The result showed no significant difference between 2 groups with RR of 1.00 (95% CI 0.99; 1.01, *p* = 0.63) (Figure 4A).

Postoperative complications were reported in 14 studies, including 1111 cases in eER and 826 in LR. Owing to the extremely low heterogeneity (*I* ^2^ = 9.2%), the common effect model was chosen for evaluation. The result showed no significant difference between the two groups, with RR of 1.02 (95% CI 0.75; 1.38, *p* = 0.89) (Figure 4B).

Postoperative infectious complications were reported in 13 studies, including 982 cases in eER and 761 in LR. Owing to the extremely low heterogeneity (*I* ^2^ = 0%), the common effect model was chosen for evaluation. The result showed no significant difference between the two groups, with RR of 0.78 (95% CI 0.44; 1.36, *p* = 0.37) (Figure 4C). Regarding completion rate, postoperative complications, and postoperative infectious complications, each of the funnel plots was symmetric, indicating no evidence of publication bias (File S5).

### 3.3 Subgroup Analysis

**FIGURE 5:**
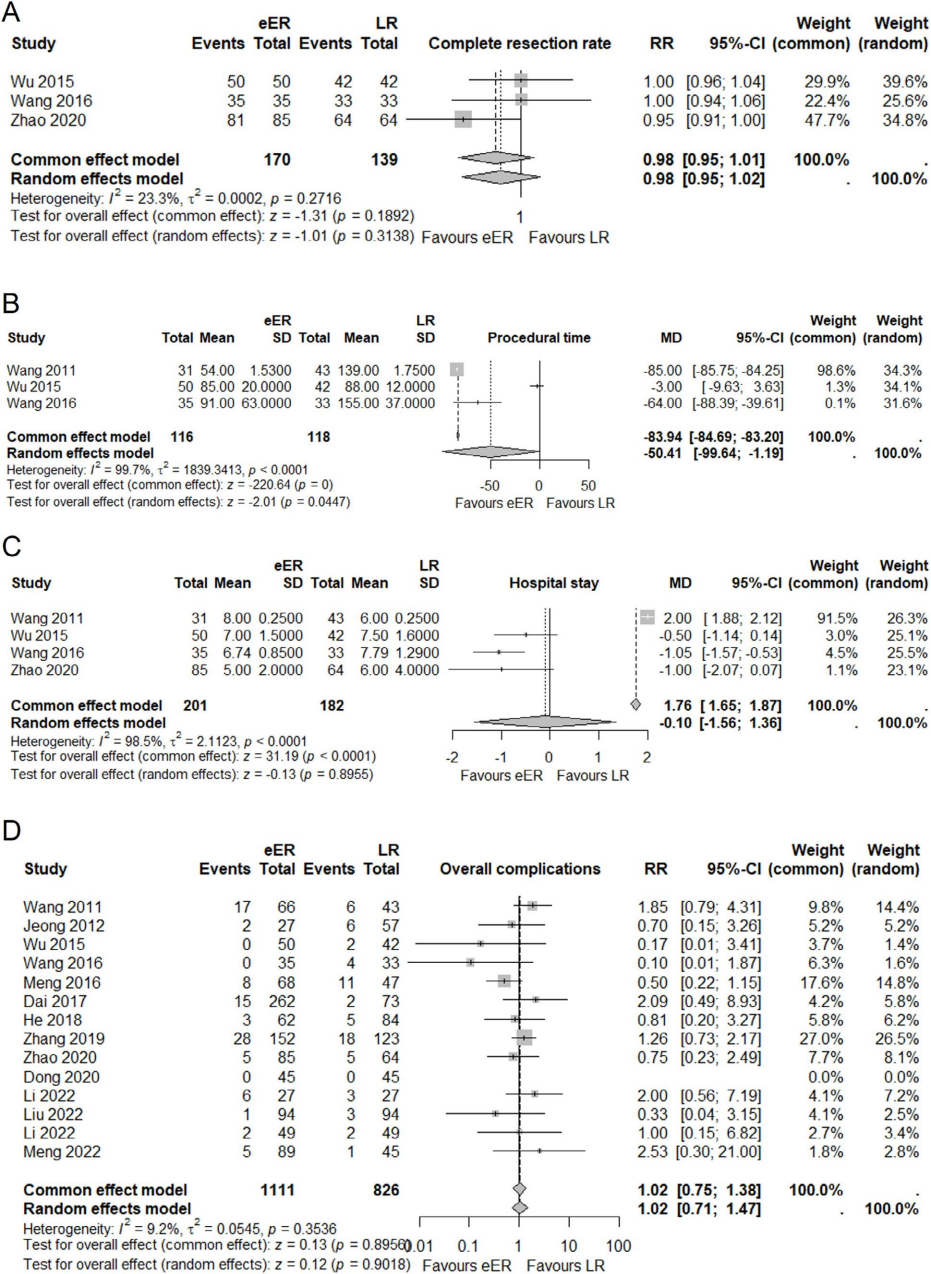
Forest plot for complete resection rate between EFTR and LR. (A) Forest plot for complete resection rate between EFTR and LR. (B) Forest plot for procedural time between EFTR and LR. (C) Forest plot for hospital stays between EFTR and LR. (D) Forest plot for overall complications between EFTR and LR.

A subgroup analysis comparing the several outcomes between EFTR and LR included three studies (170 EFTR cases, 139 LR cases) for the complete resection rate. Owing to the low heterogeneity (*I* ^2^ = 23.3%), the common effect model was chosen for evaluation. The result showed no significant difference between the 2 groups with RR of 0.98 (95% CI 0.95; 1.01, *p* = 0.18) (Figure 5A).

Procedural time was compared in three studies (116 EFTR cases, 118 LR cases). Owing to the high heterogeneity (*I* ^2^ = 99.7%), the random‐effects model was chosen for evaluation. The result showed favor in EFTR with MD of −50.41 (95% CI ‐99.64; −1.19, *p* = 0.044). (Figure 5B).

Hospital stay was compared in four studies (201 EFTR cases, 182 LR cases). Owing to the high heterogeneity (*I* ^2^ = 98.5%), the random‐effects model was chosen for evaluation. The result showed no significant difference between the 2 groups with MD of −0.10 (95% CI ‐1.56; −1.36, *p* = 0.89). (Figure 5C).

Overall complications were compared in three studies (170 EFTR cases, 139 LR cases). Owing to the low heterogeneity (*I* ^2^ = 3.8%), the common effect model was chosen for evaluation. The result showed no significant difference between the 2 groups with MD of 0.40 (95% CI 0.15; 1.06, *p* = 0.066). (Figure 5D).

The other outcomes could not be analyzed owing to insufficient extractable data across the included studies.

## 4 Discussion

In this meta‐analysis, the primary outcome, the complete resection rate, demonstrated a slight but statistically significant superiority in favor of LR. Conversely, procedural time, blood loss, time to oral intake, and hospital stay were all significantly favorable for eER. Although publication bias was detected for the complete resection rate, suggesting that the results should be interpreted with caution, nearly all included studies reported a 100% complete resection rate in LR. Therefore, the finding that LR achieves a higher complete resection rate appears to be reasonable. Furthermore, the superiority of eER in the aforementioned four outcomes supports its minimally invasive nature compared to LR. Although concerns were raised regarding the potential need for conversion to surgery, as well as an increased risk of postoperative complications in eER, no significant differences were observed in overall or infectious complication rates, nor the completion rate.

G‐SMTs are primarily concerning owing to G‐GIST; however, a wide range of other tumors, including schwannoma, leiomyoma, lipoma, neurofibroma, neuroendocrine tumors, lymphoma, and ectopic pancreas, can also be encountered. Owing to the difficulty of obtaining a definitive preoperative diagnosis, these lesions are often resected as part of a diagnostic‐therapeutic approach. Among G‐SMTs, G‐GISTs pose a unique challenge, as capsule disruption during resection carries a risk of tumor seeding [3, 30]. Therefore, avoiding intraoperative capsule rupture is of paramount importance, and complete resection without capsule damage is the fundamental principle in G‐SMTs resection. In this study, the complete resection rate was slightly higher in LR; however, when eER was limited to EFTR, the outcomes were comparable to those of LR. This finding suggests that, particularly for tumors with deep invasion, an initial strategy of EFTR may enable eER to achieve a complete resection rate that is not significantly different from that of LR. In fact, for G‐GISTs, complete resection has already been identified as a key prognostic factor contributing to long‐term survival and can thus be considered the most critical short‐term oncological outcome. Given the benefits of eER in terms of procedural time, blood loss, time to oral intake, and hospital stay, its minimal invasiveness offers significant advantages. These findings highlight the potential of eER as a promising treatment option for G‐SMTs.

Recent studies have demonstrated that eER for G‐GISTs offers superior short‐term outcomes, including reduced procedural time, shorter hospital stay, and lower blood loss [7, 8, 31]. Our meta‐analysis confirmed the previously reported short‐term advantages of eER, including a shorter time to oral intake. On the other hand, the complete resection rate remains a topic of debate among studies. A meta‐analysis by Wang et al. reported no significant difference between eER and LR, whereas Liu et al. suggested that LR may achieve a higher complete resection rate [7, 8]. In our study, LR demonstrated a slightly higher complete resection rate than eER. Furthermore, EFTR has been reported to achieve a higher complete resection rate and technical success compared to conventional eER, making it a promising option, particularly for G‐GISTs with deep muscular involvement. Recent studies have also reported that EFTR achieves a higher rate of en‐bloc complete resection compared to STER, suggesting that EFTR may offer superior oncological safety among the various eER techniques [32]. Therefore, we conducted a subgroup analysis comparing EFTR with LR by including only studies in which outcomes specific to EFTR could be extracted. The results of this meta‐analysis also support these findings, further highlighting the potential advantages of EFTR in such cases [4]. Moreover, the long‐term oncological safety of eER remains a subject of ongoing discussion. Future studies should focus on refining the optimal indications for eER through detailed subgroup analyses based on tumor location and size.

eER was originally developed as a treatment for early‐stage tumors confined to the superficial layers of the gastrointestinal tract. However, its indications have expanded with the establishment of eER, allowing for the resection of lesions that extend deeper into the submucosal layer. As eER, several advanced endoscopic techniques have been developed for the resection of G‐SMT arising from the muscularis propria layer, including EFTR, submucosal tunneling endoscopic resection (STER), and endoscopic submucosal excavation (ESE), which are included in this study. EFTR, first reported in 2008, enables en bloc resection of SMTs through full‐thickness excision while ensuring defect closure, making it a viable alternative to surgical resection, particularly for anatomically challenging locations [33]. STER, introduced in 2012, utilizes a submucosal tunneling approach to excise tumors while preserving the overlying mucosa, thereby reducing the risk of complications such as perforation and stricture [34]. This technique is particularly suitable for SMTs of the stomach, especially those located in the antrum or body, where traditional surgical resection may pose greater challenges [35]. ESE involves direct dissection within the submucosal layer to enucleate SMTs while minimizing damage to surrounding tissue, achieving high complete resection rates but facing technical limitations in cases of deep invasion [36]. These evolving techniques have demonstrated favorable outcomes in selected cases, and their application continues to expand based on tumor characteristics, anatomical location, and operator expertise. However, while there are several reports comparing eER with LR as a treatment for G‐SMTs, sufficient evidence has not yet been established to provide a definitive conclusion. Therefore, this meta‐analysis was conducted to establish the current evidence for this comparison.

This study aimed to comprehensively include all potentially relevant studies comparing the outcomes of eER and LR for G‐SMTs. To achieve this, we did not restrict the inclusion criteria to G‐GISTs alone but included all benign G‐SMTs without a definitive preoperative diagnosis. As a result, this meta‐analysis incorporated a larger dataset than previous meta‐analyses that focused solely on G‐GISTs, and to the best of our knowledge, it represents the largest meta‐analysis to date comparing treatment strategies for G‐SMTs [8, 31, 37, 38, 39, 40, 41]. Expanding the study population to include all G‐SMTs did not lead to an increase in overall heterogeneity. However, similar to previous meta‐analyses for G‐GISTs, heterogeneity arising from tumor location, size, and the diversity of treatment modalities remained a methodological challenge. To minimize bias, the data included for each outcome were carefully reviewed by multiple independent reviewers, ensuring that inappropriate data were excluded. Additionally, Baujat plot analysis and linear regression tests for funnel plot asymmetry were conducted to assess the potential presence of publication bias. Despite these efforts, it was not feasible to categorize individual studies based on tumor size and location, and thus, some degree of heterogeneity remains inherent to the analysis. This limitation must be considered when interpreting the results. Especially, while our meta‐analysis demonstrated a statistically significant difference favoring LR in terms of the complete resection rate, sensitivity analysis revealed that a single study had a substantial impact on the findings. Excluding this study resulted in the loss of statistical significance, underscoring its strong influence on the overall results. Furthermore, the presence of publication bias was suggested (Egger's test, *p* = 0.0381), warranting cautious interpretation of our findings. However, as the included study represents a clinically meaningful dataset, we opted to retain it in the primary analysis while acknowledging this limitation.

eER has the potential to become a standard treatment option for G‐SMTs, given its clear minimally invasive nature, resulting in favorable intraoperative and short‐term postoperative outcomes compared to LR, as demonstrated in this meta‐analysis. However, while several studies have reported on the feasibility and safety of eER, concerns remain regarding the technical difficulty of the procedure and the risk of intraoperative complications necessitating conversion to surgery [1, 4, 42, 43]. Therefore, the widespread adoption of eER should be approached with caution. The primary challenges with eER, compared to LR, are the difficulty of maneuverability and the lack of auxiliary instruments to create counter‐traction. In rectal surgery, Transanal Endoscopic Microsurgery (TEM), introduced by Gerhard Buess in 1983, represents an intermediate approach between LR and eER and is widely practiced in Western countries owing to its effectiveness [44]. TEM is an endoluminal surgery utilizing a rigid proctoscope, enabling precise and dexterous resection of rectal tumors [45]. In the upper gastrointestinal tract, the distance from the natural orifice to the target tumor is longer and more complex compared to the rectum, making the application of TEM using rigid endoscopes challenging. However, in recent years, increasing attention has been given to the potential of endoluminal robotic surgery, with several research studies already underway [46, 47, 48]. Future innovations in endoluminal robotic systems may significantly lower the technical barriers associated with eER, making it a more accessible and feasible approach for G‐SMTs resection. Further advancements in this area are expected to enhance widespread clinical adoption.

This study has several limitations. First, as mentioned earlier, eER encompasses various techniques, including endoscopic submucosal dissection (ESD), EFTR, STER, and ESE. Similarly, LR includes a wide range of procedures, such as stapled or hand‐sewn wedge resection, distal gastrectomy, proximal gastrectomy, and intragastric surgery. Despite the broad inclusion criteria applied in this study to analyze a large number of publications, the available data were insufficient to perform a uniform analysis considering all these variations. Likewise, tumor location and size may have introduced bias. However, since this current study focused exclusively on eER and LR as treatment modalities, the mean or median tumor size reported in the included studies was ≤ 5 cm, suggesting that there was no substantial variation in tumor size across the analyzed population. Furthermore, regarding tumor location, previous reports have indicated that eER is technically challenging in various anatomical sites, such as the fundus owing to its thin gastric wall, the cardia owing to limited accessibility, and the lesser curvature or posterior wall owing to endoscopic maneuverability constraints [49, 50]. However, the relationship between tumor location and the technical difficulty of eER remains inadequately elucidated and warrants further investigation. Notably, none of the studies included in this meta‐analysis were randomized controlled trials, and it is possible that the choice of resection method was influenced by the physician's discretion based on tumor location. This potential source of selection bias should be carefully considered when interpreting the results. Second, while cost analysis would have been a valuable comparison, the limited number of studies reporting cost‐related outcomes made it difficult to conduct a meaningful analysis. Further research on this topic is warranted to provide more comprehensive insights. Third, all studies included in this meta‐analysis were retrospective. However, compared to the meta‐analysis by Wang et al., which included a single propensity score‐matching (PSM) study on gastric GISTs, our analysis incorporated three PSM studies, suggesting a slight improvement in the overall quality of the included evidence [7]. Nevertheless, further accumulation of high‐quality evidence, particularly through randomized controlled trials (RCTs), is necessary to strengthen the findings in this field. Moreover, this meta‐analysis was primarily based on retrospective studies with relatively small sample sizes, and all analyses were conducted using tabulated data without access to individual patient‐level data. Therefore, the findings should be interpreted with caution owing to the inherent limitations of the available evidence. Lastly, it should be noted that most of the included studies did not report long‐term oncological outcomes such as recurrence or disease‐free survival. As these endpoints are essential to fully establish the oncological validity of eER and EFTR, especially in potentially malignant lesions like G‐GISTs, the absence of such data limits the interpretation of the long‐term safety of these approaches.

In conclusion, this meta‐analysis demonstrated that while LR may have a potential advantage over eER in terms of complete resection rate, eER showed superiority in procedural time, blood loss, time to oral intake, and hospital stay, highlighting its minimally invasive benefits. Furthermore, when eER was limited to EFTR, the complete resection rate was comparable to that of LR. Although the widespread adoption of EFTR still faces challenges, further refinement of techniques and the development of endoluminal surgical robots may facilitate its standardization and broader clinical implementation in the future.

## Author Contributions

Kengo Hayashi, Saki Hayashi, and Noriyuki Inaki contributed to the study concept and design, acquisition, analysis, and interpretation of data. Data collection was conducted by, Kengo Hayashi, Saki Hayashi, Chiara Meroni, Chiara Marafante, Rebecca Dallorto, and Carlo Alberto Ammirati, whereas, Roberto Passera performed the statistical analyses. Kengo Hayashi drafted the manuscript. Noriyuki Inaki and Alberto Arezzo, supervised the study. All authors read and approved of the final manuscript.

## Ethics Statement

This study is based entirely on previously published research and did not involve any new studies with human participants or animals conducted by the authors. Therefore, approval from an ethics committee was not required, and the acquisition of informed consent was not necessary.

## Conflicts of Interest

Dr. Noriyuki Inaki is an Editorial Board member of ASES Journal and a co‐author of this article. To minimize bias, they were excluded from all editorial decision‐making related to the acceptance of this article for publication.

## Supporting information


**File S1.** PRISMA 2020 checklist.

**File S2.** Newcastle‐Ottawa Scale.

**File S3A.** Linear regression test of funnel plot asymmetry for the analysis of complete resection rate. **File 3B:** Baujat plot for the analysis of complete resection rate.

**File S4.** Funnel plot. A: Procedural time, B: Blood loss, C: Time to oral intake, D: Hospital stay.

**File S5.** Baujat plot. A: Completion rate, B: Overall complication rate, C: Infectious complication rate.

## Data Availability

The data that support the findings of this study are available from the corresponding author upon reasonable request.
