# Supplementary material for: Endoscopic Resection Versus Laparoscopic Resection for Gastric Submucosal Tumors: A Systematic Review and Meta‐Analysis of Safety and Efficacy
Source: Asian J Endosc Surg. 2025 Jun 24;18(1):e70104. doi: 10.1111/ases.70104 (PMC12187583; doi:10.1111/ases.70104)
Supplement: Supplementary file 1 — File S1. PRISMA 2020 checklist. [file ASES-18-e70104-s001.docx]

PRISMA 2020 Checklist

| Section and Topic | Item # | Checklist item | Location where item is reported |
| --- | --- | --- | --- |
| TITLE | 1 | Identify the report as a systematic review. | Title page |
| ABSTRACT | 2 | See the PRISMA 2020 for Abstracts checklist. | Page 2(Abstract) |
| INTRODUCTION | 3 | Describe the rationale for the review. | Introduction, Page 4 |
|  | 4 | Provide an explicit statement of the objective(s) the review addresses. | Introduction, end of Page |
| METHODS | 5 | Specify inclusion/exclusion criteria and study grouping. | Methods, 'Eligibility Criteria' |
|  | 6 | Information sources (e.g., databases, registers). | Methods, ' Literature search strategy' |
|  | 7 | Search strategy (full strategies for all databases). | Methods, 'Literature search strategy' |
|  | 8 | Selection process (e.g., number of reviewers). | Methods, 'Literature screening and data extraction' |
|  | 9 | Data collection process. | Methods, ' Literature screening and data extraction ' |
|  | 10 | Data items (outcomes and variables). | Methods, ' Literature screening and data extraction ' |
|  | 11 | Risk of bias assessment. | Methods, 'Quality Assessment' |
|  | 12 | Effect measures (e.g., RR, OR). | Methods, 'Statistical Analysis' |
|  | 13 | Synthesis methods. | Methods, 'Statistical Analysis' |
| RESULTS | 16 | Flow of studies through the review. | Results |
|  | 17 | Study characteristics. | Results, Table 1 |
|  | 18 | Risk of bias in studies. | Results |
|  | 19 | Results of individual studies. | Results |
|  | 20 | Results of syntheses (pooled analyses). | Results |
| DISCUSSION | 23 | Interpretation of results in context. | Discussion, Pages 14-20 |
|  | 24 | Limitations of evidence and review. | Discussion, Pages 21 |
| OTHER INFORMATION | 27 | Availability of data, code and materials. | Available on request |
