# Supplementary figures and images for: Endoscopic Resection Versus Laparoscopic Resection for Gastric Submucosal Tumors: A Systematic Review and Meta‐Analysis of Safety and Efficacy
Source: Asian J Endosc Surg. 2025 Jun 24;18(1):e70104. doi: 10.1111/ases.70104 (PMC12187583; doi:10.1111/ases.70104)

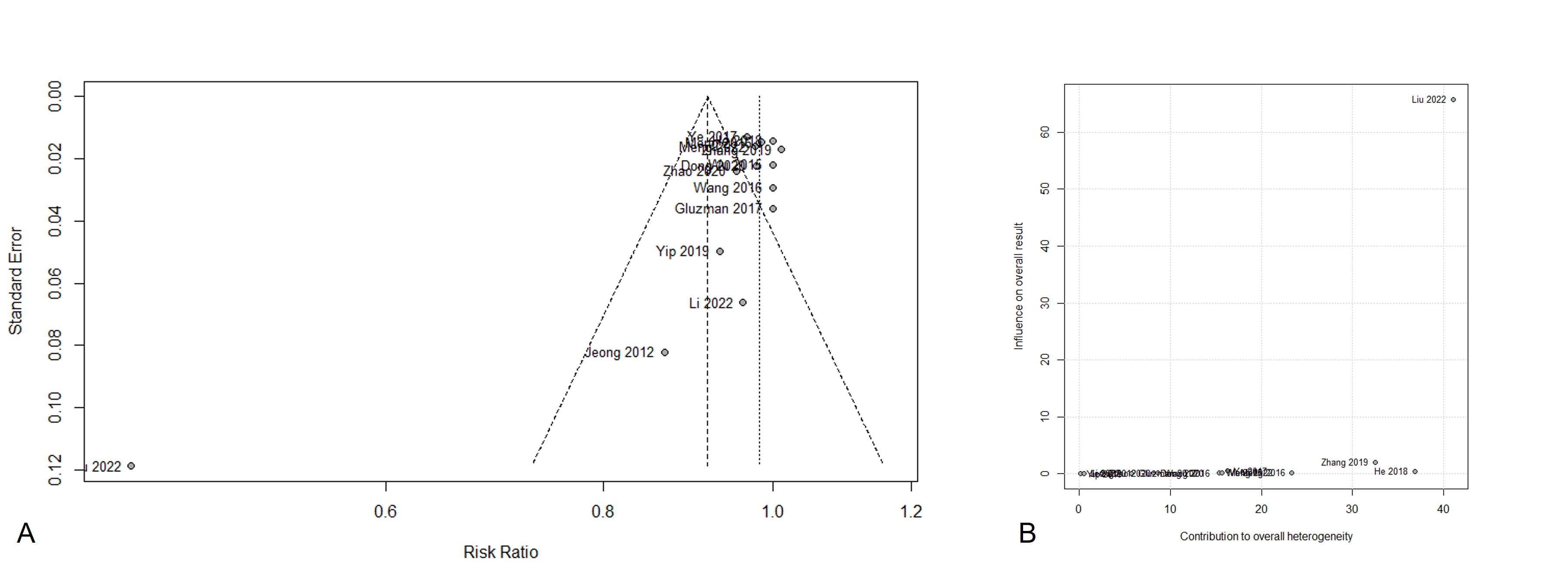

Supplement: Supplementary file 3 — File S3A. Linear regression test of funnel plot asymmetry for the analysis of complete resection rate. File 3B: Baujat plot for the analysis of complete resection rate. [file ASES-18-e70104-s005.jpg]

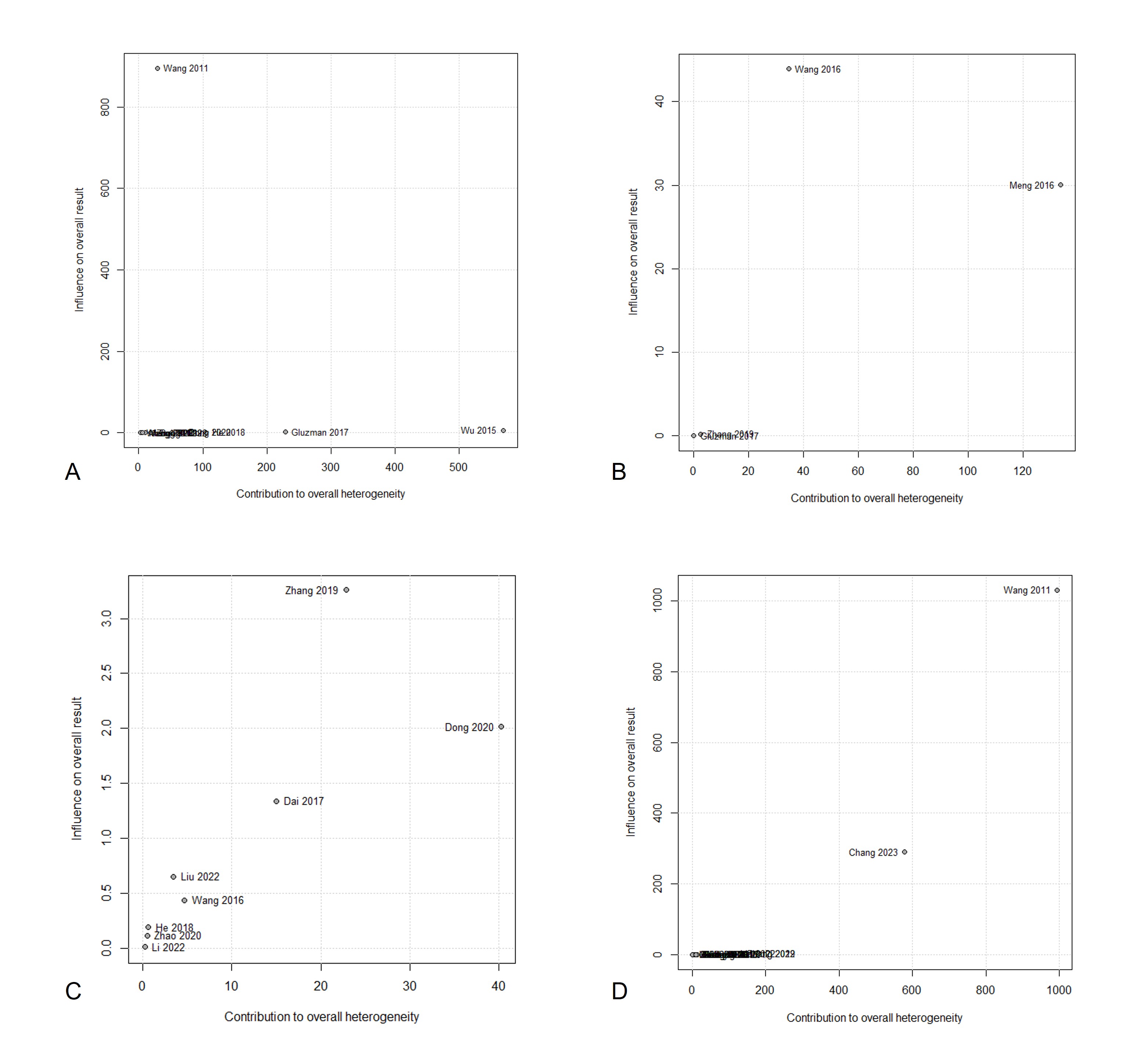

Supplement: Supplementary file 4 — File S4. Funnel plot. A: Procedural time, B: Blood loss, C: Time to oral intake, D: Hospital stay. [file ASES-18-e70104-s002.jpg]

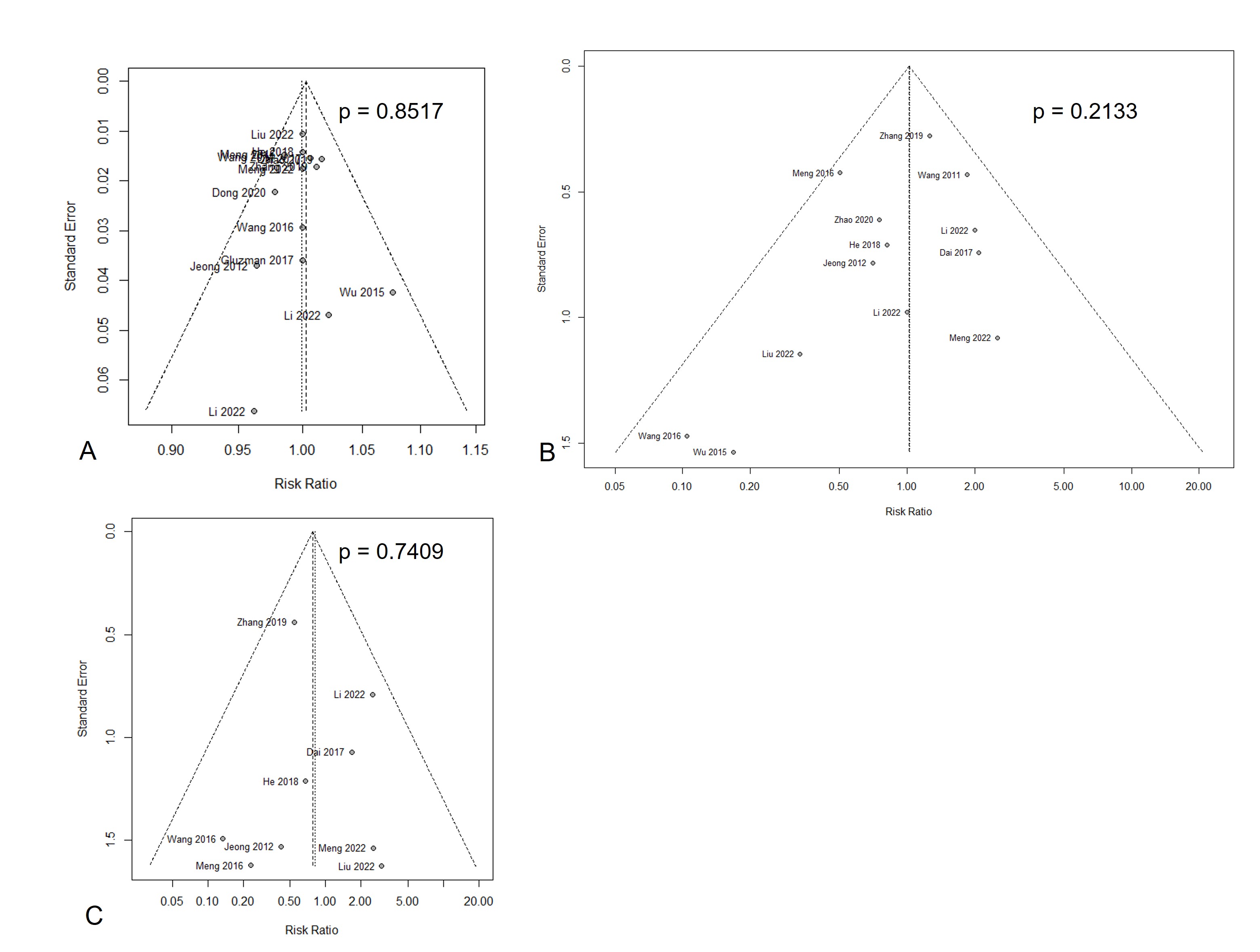

Supplement: Supplementary file 5 — File S5. Baujat plot. A: Completion rate, B: Overall complication rate, C: Infectious complication rate. [file ASES-18-e70104-s004.jpg]
